# CK2.3, a Mimetic Peptide of the BMP Type I Receptor, Increases Activity in Osteoblasts over BMP2

**DOI:** 10.3390/ijms20235877

**Published:** 2019-11-23

**Authors:** Hilary Weidner, Victor Yuan Gao, Debra Dibert, Sean McTague, Mark Eskander, Randall Duncan, Liyun Wang, Anja Nohe

**Affiliations:** 1 Department of Biological Sciences, University of Delaware, Newark, DE 19716, USA; 2 Department of Mechanical Engineering, University of Delaware, Newark, DE 19716, USA; vyg@udel.edu (V.Y.G.); lywang@udel.edu (L.W.); 3 Christiana Care Hospital, Newark, DE 19716, USA; ddibert@christianacare.org (D.D.); smctague@christianacare.org (S.M.); markeskander77@gmail.com (M.E.); 4 Department of Biology, University of Michigan, Flint, MI 48502, USA; duncanra@umflint.edu

**Keywords:** osteoporosis, femoral head, osteoarthritis, BMP2, CK2.3, BMD

## Abstract

Bone is one of the most important organs in the human body. It provides structure, function, and protection for other vital organs; therefore, bone maintenance and homeostasis are critical processes. As humans age, their bone mineral density decreases, which leads to diseases like osteoporosis. This disease affects one in two women and one in five men aged 50 and over. As the aging population increases, the interest and significance of studying this debilitating bone disease becomes more relevant. Current therapeutic products for osteoporosis have many side effects and can be taken for a limited number of years. Most therapeutic products only focus on decreasing bone resorption, not increasing bone formation. Bone morphogenetic protein 2 is an essential growth factor that drives osteoblast differentiation and activity and is essential for bone formation. However, usage in the clinic is unsuccessful due to several side effects. Recently, a signaling disparity in bone marrow stromal cells within the bone morphogenetic protein pathway that led to decreased bone morphogenetic protein 2 responsiveness was identified in patients diagnosed with osteoporosis. However, it is unclear how other cell populations, especially osteoblasts, which are key players in bone remodeling, are affected and whether the bone morphogenetic protein pathway is affected during osteoporosis. Our research group designed a novel peptide, casein kinase 2.3, that acts downstream of the bone morphogenetic receptor type Ia and increases bone mineralization in murine cells and primary bovine osteoblasts. The aim of the study presented here was to compare the responsiveness of osteoblasts to bone morphogenetic protein 2 and casein kinase 2.3, especially in patients diagnosed with osteoporosis. Mature osteoblasts were extracted from patients diagnosed with osteoporosis or osteoarthritis from Christiana Care Hospital in Newark, Delaware. They were stimulated with either bone morphogenetic protein 2 or casein kinase 2.3, and their effect on osteoblast activity was determined. The osteoporotic patients showed no mineralization response to bone morphogenetic protein 2 stimulation, while the osteoarthritis patients significantly responded to bone morphogenetic protein 2 stimulation. Furthermore, markers for osteoblast activity were increased by casein kinase 2.3, which was in sharp contrast to bone morphogenetic protein 2. This further supports a major bone morphogenetic protein signaling disparity in both the elderly and those suffering with osteoporosis. Both patient types did significantly respond to casein kinase 2.3. Further analysis of the bone morphogenetic protein pathway could lead to new therapeutic products for osteoporosis.

## 1. Introduction

Bone is one of the most important organs in the human body. It provides a stabile structure as well as protection of other vital organs and is critical for mobility. Recently, it was discovered that bone can be classified as an endocrine organ due to the storage and circulation of calcium, phosphate, and osteocalcin. It is an extremely dynamic organ as it is constantly being remodeled by a specialized group of cells. These cells, namely, osteoblasts (bone building), osteoclasts (bone resorbing), and osteocytes, are responsible for continued maintenance of the skeletal systems [1]. Under normal conditions, there is a balance between the osteoblasts and osteoclasts; however, as humans age, the distribution between osteoblast and osteoclast activity becomes unhinged [2]. Less osteoblast activity or increased osteoclast activity leads to more porous or brittle bone. Osteoporosis (OP) is a bone disease characterized by low bone density, which can lead to decreased bone strength and an increased risk of fracture. It is the most common disease in humans [3]. The deterioration of bone tissue microarchitecture equates to a decrease in overall bone density, and measuring bone mineral density (BMD) can approximate the relative skeletal mass of the bone tissue. BMD refers to, and measures, the mineralized bone [4,5]. OP primarily affects men and women over the age of 50 due to decreased efficiency in the bone remodeling cycle (bone turnover). Most osteoporotic fractures occur in the spine, hip, or knee [4]. Hip fractures are the most severe and result from a fall or occur spontaneously [6,7]. They are extremely painful, require hospitalization for an average of 30 days [8,9], and have an increased rate of morbidity [10]. Most treatments for osteoporosis focus on decreasing osteoclast activity (antiresorptive treatments). There are a limited number of treatments available that focus on increasing osteoblast activity (anabolic treatments). Those that are available to OP patients can only be taken for a limited number of years and have been associated with osteosarcoma. Prevention is the best treatment for osteoporosis, but this does not help the 200 million already diagnosed with OP; therefore, there is a need to provide better therapeutic products. Another prevalent skeletal disease is osteoarthritis (OA), also known as degenerative joint disease. In OA, cartilage is greatly diminished in the knee, hip, hands, feet, and spine [11]. There are currently no medications on the market that reverse the deterioration of cartilage; instead, they focus on decreasing the pain or inflammation [12]. In order to find successful treatments, understanding the underlying mechanisms of this disease is crucial [13].

Bone morphogenetic protein 2 (BMP2) is the most critical growth factor for osteoblast differentiation and activity. Without BMP2, bone would not be able to be formed; therefore, it is a critical growth factor to be studied. It has been studied in various mouse models and has been shown to be an extremely effective bone-promoting growth factor [14]. There are, however, drawbacks when using mouse models. While the mouse system is similar to a human system, it is not exact. In fact, many successful cancer studies that have focused on the mouse model have repeatedly failed in clinical trials [15]. There is less than 8% successful translation rate of using a mouse model to human clinical trials [15]. Simply put, a successful mouse study does not equate to a successful human study, and BMP2 is unfortunately no exception. It is approved by the Food and Drug Administration (FDA) for the short-term healing of long-bone fractures [16]; however, long-term usage of BMP2 increases osteoclast activity, which also increases the patients’ fracture risk [17]. There are a multitude of side effects that have been noted after utilizing BMP2 in this manner, namely, ectopic bone formation, hematoma, seroma, and extended or permanent nerve pain or damage. There are no clear advantages of BMP2 use for the treatment of long-bone fractures [18], spinal fusions [19], or OP. While BMP2 itself may not be a viable treatment, the exploitation of the BMP pathway is of interest because it induces important skeletal cells and is a necessity for osteoblast differentiation. Differing levels of BMP2 dictate the differentiation pathway of bone cells; therefore, studying the BMP pathway is crucial. In addition to the importance of BMP2 development, recent research has shown a potential BMP signaling disparity in those diagnosed with osteoporosis [20]. Some groups have observed a decrease in BMP2-activated BMP canonical signaling in primary bone marrow stromal cells (BMSCs), which is a possible explanation for the decrease in bone formation observed in osteoporosis [20].

Taking a deeper look into the BMP2-activated pathway, BMP2 activates the pathway by binding to two receptors: bone morphogenetic protein receptor type Ia (BMPRIa) and bone morphogenetic protein receptor type II (BMPRII). BMPRII then phosphorylates BMPRIa at three separate locations, and an interacting protein called casein kinase 2 (CK2) is released. CK2 signals to induce osteoblastogeneis, adipogenesis, osteoclastogenesis, and chondrogenesis [21]. A mimetic peptide, CK2.3, binds to CK2, which blocks its interaction with BMPRIa at a specific phosphorylation site [22,23]. Stimulation of osteoblast and osteoblast precursor cells with CK2.3 was found to significantly increase mineralization, which is indicative of preliminary bone formation. Furthermore, CK2.3 increased osteoblast activity in extracted calvarial and extracted BMSCs. Injection of CK2.3 via tail veins of mice led to increased trabecular BMD. Mineralization apposition rate (MAR), another measure for bone formation, was also significantly higher in mice injected with CK2.3. [24].

As mentioned previously, following a mouse model can be misleading, especially when trying to relate the findings from a mouse to a human. Therefore, in this study, we wanted to observe the effects of BMP2 and CK2.3 directly on isolated cells from humans. To do this, we obtained human femoral heads from Christiana Care Hospital in Newark, Delaware, from patients diagnosed with either osteoporosis (POP) or osteoarthritis (POA). Mature osteoblasts were isolated from the patients’ femoral heads. There is not a lot of current research available on mature osteoblasts directly isolated from human patients. Most studies focus on BMSCs because they are easy to isolate and culture. Mature osteoblasts require several weeks of culture before they are ready for plating and fixation, which makes this study novel. We hypothesized that cells isolated from both POP and POA would have a positive mineralization response to both BMP2 and CK2.3, as our lab’s previous results have indicated. BMP2 was used as a positive control because it is a stimulant that pushes MSCs deeper into the osteoblast lineage, causing them to mineralize. Only POA showed a positive mineralization response to both BMP2 and CK2.3 stimulations. Cells from POP did not respond to BMP2 stimulation; they only responded to CK2.3 stimulation. This is very similar to previous research on BMSCs from POP. We have shown for the first time that mature osteoblasts extracted from POP do not respond to BMP2 stimulation. This is indicative of a potential signaling discrepancy in osteoporosis, and further research could highlight potential therapeutic targets to regulate the bone remodeling cycle in osteoporosis.

## 2. Results

### 2.1. Femoral Heads Isolated from POP Had Lower BMD Compared to Femoral Heads Isolated from POA

**Figure 1:**
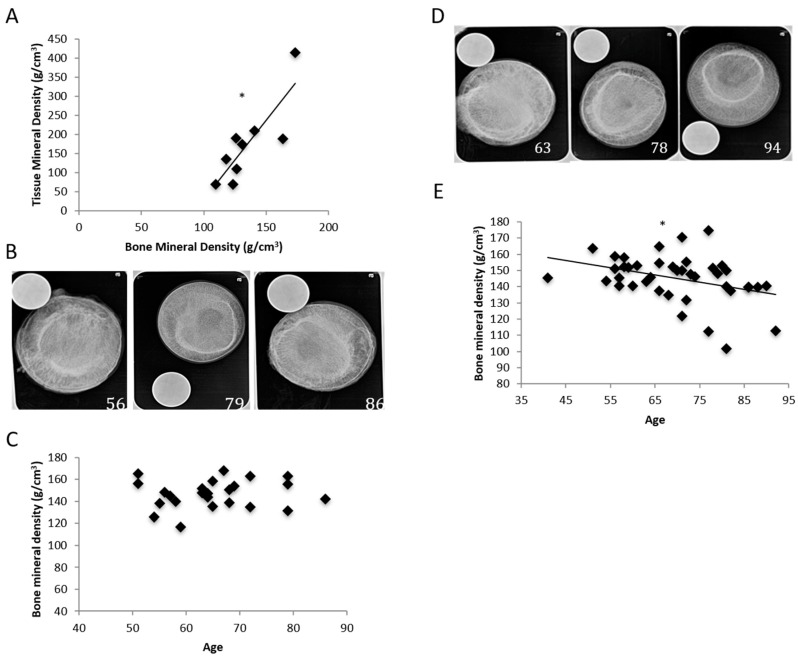
Tissue mineral density (TMD) measurements were calculated in order to determine and validate the bone mineral density (BMD) measurements and the corresponding trends observed with the BMD measurements. (**A**) A positive correlation was observed when comparing the microcomputed tomography (microCT) TMD measurements to the BMD measurements. Femoral necks of a total of 10 randomized patients diagnosed with osteoporosis (POP) were scanned through microCT in order to generate the computed TMD measurements. These measurements were then directly compared with the corresponding BMD measurements in order to validate any potential trends seen with BMD and age. (**B**) X-ray images of three femoral heads extracted from patients diagnosed with osteoarthritis (POA). All patients were female (age is labeled). X-rays were taken down the midsagittal plane, with a penny used to verify consistency in distance between the handheld X-ray (Nomad Pro Handheld X-ray system) and specimen. (**C**) We used single-photon absorptiometry (SPA), which utilizes a single-energy photon beam that passes through the bone to a detector, in order to quantify patients’ respective BMD. Femoral heads of 25 female POA were X-rayed, and their BMD was quantified. The data was then plotted and compared to increasing age, and trends were observed. (**D**) Radiographs of the extracted femoral heads of three female POP (age is labeled). The X-rays were taken down the midsagittal plane, with a penny used to determine the distance between the X-ray machine and femoral head. (**E**) The extracted femoral heads were X-rayed, and their BMD was quantified using SPA. All specimens were extracted from female patients, and 42 X-rays were quantified in total. * denotes significant correlation (*p* < 0.05).

Single-photon absorptiometry (SPA) was used to measure BMD instead of microcomputed tomography (microCT) due to time and cost constraints. In order to verify if our calculated BMD data was representative of our samples, tissue mineral density (TMD) was calculated from the femoral necks of randomized patients. TMD and BMD values from 12 osteoporotic females, aged from 56 to 86, were calculated and used. TMD was determined through a microCT scan, and all measurements are in mm Hg/cm. The TMD values were compared to the BMD values of the same patients, and a positive correlation was observed (Figure 1A).

We obtained radiographs of each femoral head extracted from POA. Representative radiographs are shown in Figure 1B. BMDs of a total of 25 female POA were analyzed and quantified. No decrease or increase of BMD was observed (Figure 1C). Therefore, we found no correlation between age and BMD in POA. We obtained 42 femoral heads from POP. They were X-rayed, and their bone density was quantified. We found a negative correlation between age and BMD in POP. Representative radiographs are shown in Figure 1D, and a graph of the BMD can be seen in Figure 1E. This confirms that the femoral heads were osteoporotic.

### 2.2. Cells Isolated by Digestion of Bone Are Mature Osteoblasts

**Figure 2:**
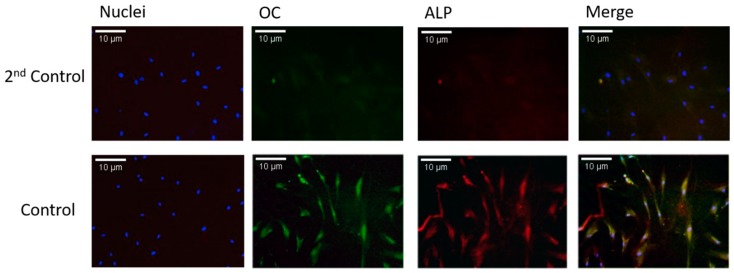
Osteoblast phenotype confirmed in isolated cells. Extracted cells were immunfluorescently stained for two osteoblast markers—alkaline phosphatase (ALP) and osteocalcin (OC)—in order to determine and confirm the cells were osteoblasts. Cells were not stimulated with either BMP2 or CK2.3.

Most studies in the bone biology field have used human primary MSCs rather than mature bone cells. This is because MSCs are extremely easy to isolate and grow. They also have a faster doubling time and thus a faster experimental turnaround time. However, there are some caveats to using MSCs. Because they are being grown and differentiated in vitro, there could be some discrepancies between how the cells react in culture versus how they would have reacted in vivo. Isolating and extracting mature osteoblasts is extremely time-consuming and difficult; however, working with mature osteoblasts is more rewarding. It is more indicative of osteoblasts’ response in vivo. In order to confirm that mature osteoblasts could be isolated from human femoral heads, the isolated cells were stained fluorescently for two osteoblast markers: alkaline phosphatase (ALP) and osteocalcin (OC). ALP is an early-stage osteoblast marker, and OC is a late-stage osteoblast marker. Unstimulated cells were stained positive for both markers (except the negative controls, i.e., 2nd control), as can be seen in Figure 2. This confirmed that approximately 90% or more positively stained cells were osteoblasts and that mature osteoblasts could be isolated from the femoral heads.

### 2.3. Higher Basal Level Mineralization in POA Compared to POP

**Figure 3:**
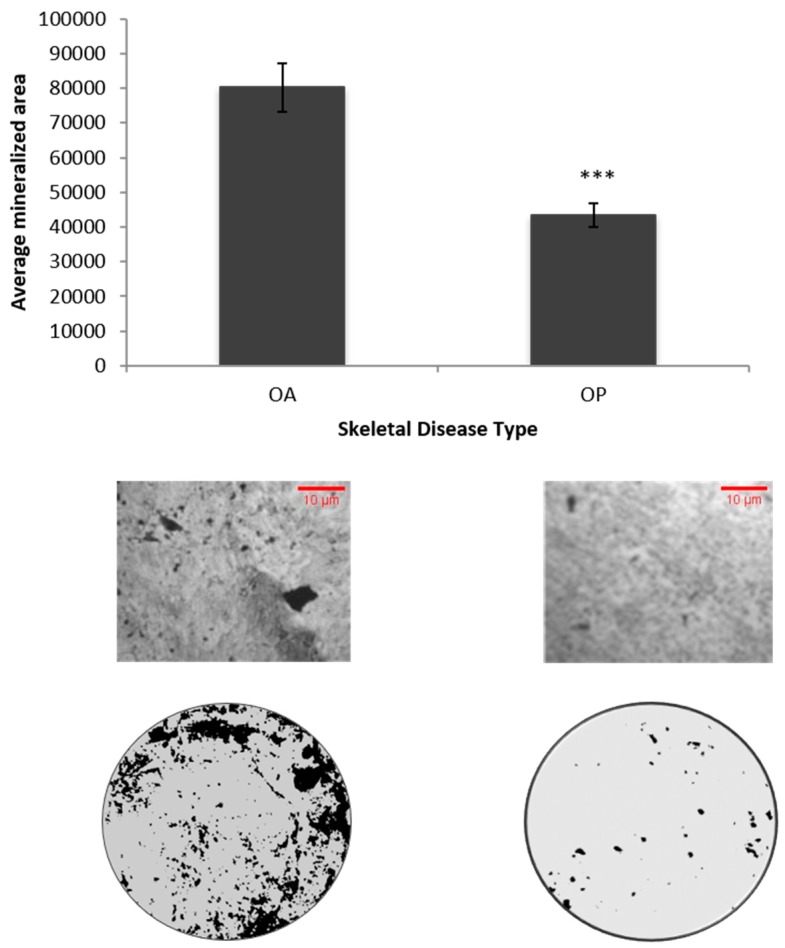
Basal level mineralization was higher in primary cells extracted from POA than primary cells extracted from POP. A von Kossa assay was used to determine mineralization potential between cell populations with the two skeletal diseases. *** denotes *p* < 0.001.

Osteoblastic cells isolated from POA have a decreased mineralization compared to cells isolated from normal patients. However, upon BMP2 stimulation, the mineralization has been shown to increase in OA osteoblasts, albeit not more than normal isolated osteoblasts [25]. We wanted to observe the mineralization potential of control or unstimulated cells from both POA and POP. The cells isolated from POA aged 54–66 had a significant amount of mineralization deposits compared with the cells isolated from POP aged 60–82 (Figure 3). These data indicate that cells from POA had a significantly higher basal mineralization level compared to POP and suggest that they have different mineralization responses to BMP2.

### 2.4. Cells Isolated from POP Show No Response to BMP2 Stimulation

**Figure 4:**
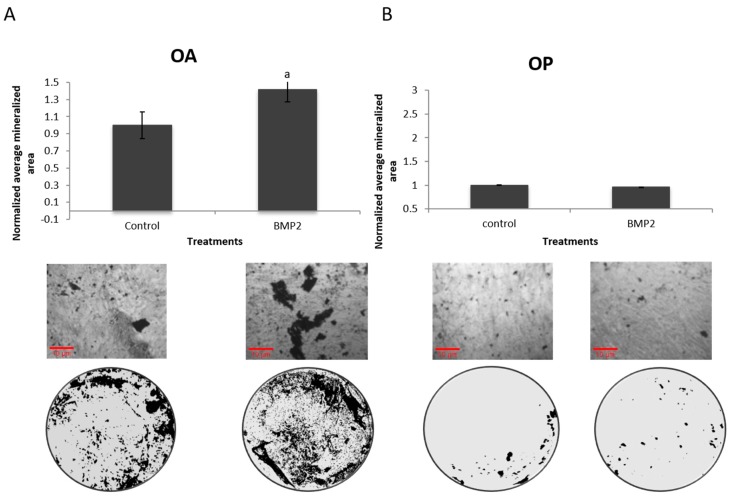
Cells isolated from POP did not respond to BMP2 stimulation, while POA did. (**A**) Five female patients were analyzed. Cells from all patients responded significantly to BMP2 compared to unstimulated cells. (**B**) Five other female patients diagnosed with osteoporosis were analyzed, and their cells showed no mineralization response compared to unstimulated cells (*p* < 0.05). a = statistically significant from control.

C2C12 cells, primary BMSCs, and primary murine osteoblasts have been found to respond to BMP2 stimulation by increasing their mineralization [24]. However, the studied cells were all from mice, and it is unclear if mature human osteoblasts would have a similar response. A von Kossa assay was performed on the extracted osteoblastic cells from five female POA and five female POP in order to assess mineralization. The cells isolated from POA responded significantly when stimulated with BMP2 (Figure 4A), while POP showed no mineralization response with BMP2 stimulation compared to the control (Figure 4B). This indicates a major signaling disparity in the BMP pathway.

### 2.5. Cells Isolated from POP Respond to Only CK2.3

**Figure 5:**
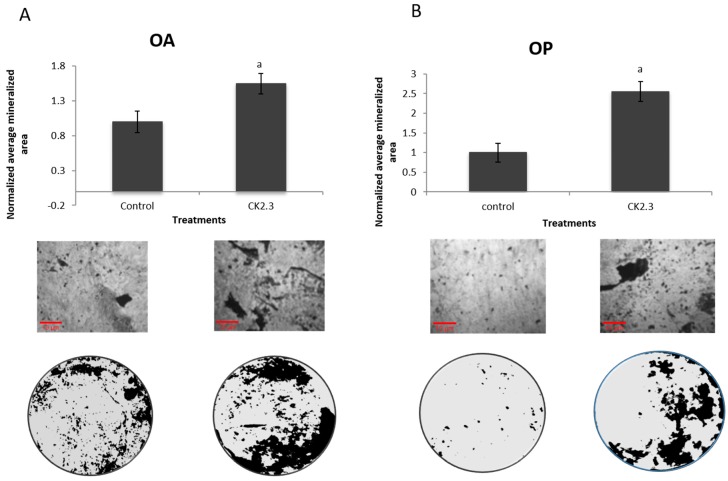
Cells isolated from both POA and POP responded to CK2.3 stimulation. (**A**) Cells from five female POA were analyzed, and significantly responded to CK2.3 stimulation compared to unstimulated or control cells. (**B**) Cells extracted from five female POP were analyzed, and all patients significantly responded to CK2.3 treatment compared to the control. CK2.3 could be a potential therapeutic product for osteoporosis (*p* < 0.05). a = statistically significant from control.

A von Kossa assay was also performed on the extracted osteoblasts from five female POA and five female POP to assess mineralization potential. The cells isolated from both POA and POP significantly responded to CK2.3 stimulation (Figure 5A,B). CK2.3 has already been shown to significantly increase mineralization in C2C12 cells, BMSCs, and primary osteoblasts isolated from rodents [24], and this study now confirms the same applies to primary osteoblasts from both POA and POP. While only the POA responded to BMP2 stimulation, both cell populations responded to CK2.3 stimulation, showing that CK2.3 has an advantage over BMP2 by increasing osteoblast activity in cells from POP.

### 2.6. CK2.3 Stimulation Increases Expression of Both Osteocalcin and Alkaline Phosphatase in Primary Human Osteoblasts

**Figure 6:**
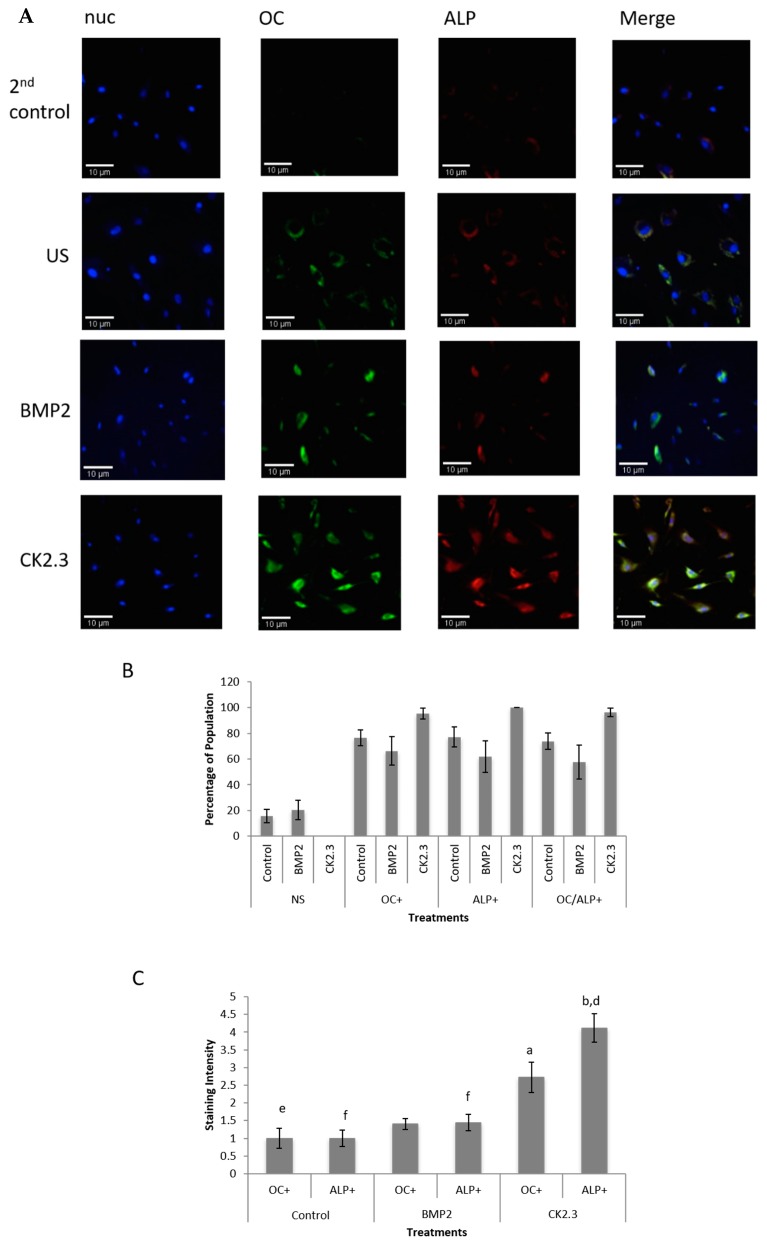
CK2.3 stimulation increased expression of both OC and ALP in primary human osteoblasts. (**A**) Cells were fluorescently stained for the presence of osteoblast markers after being stimulated with BMP2 or CK2.3 or left unstimulated (US or control). (**B**) Cells stained for OC, ALP, or both were counted and then divided by the number of nuclei in order to obtain a percentage of stained cells. All treatments resulted in an osteoblast phenotype. (**C**) The intensity of OC, ALP, or both was calculated through the “Color Histogram” function of ImageJ. CK2.3 had a significantly increased intensity of both OC and ALP compared to BMP2 and control (*p* < 0.05). a = statistically significant from Control OC+, b = statistically significant from Control ALP+, d = statistically significant from BMP2 ALP+, e = statistically significant from CK2.3 OC+, f = statistically significant from CK2.3 ALP+.

In order to observe the effects of BMP2 and CK2.3 stimulation on osteoblast expression in the cell population extracted from human femoral heads, isolated cells from three female POP were fluorescently stained for the osteoblast markers ALP and OC as well as for the nucleus (nuc). In all treatments, including control, cells stained positive for both markers (except the negative controls, i.e., 2nd control), which can be seen in Figure 6A. Cells were also counted for visible OC stain, ALP stain, both stains, or no stain (NS) in order to confirm equal seeding and population density. This was completed for cells stimulated with BMP2 or CK2.3 or cells left unstimulated (US or control). The counts were divided by the nuclei count and multiplied by 100 in order to determine the percentage of stained cells (Figure 6B). Lastly, the intensity of the stains was quantified using ImageJ in order to determine whether treatments induced a stronger expression of OC or ALP. CK2.3 had significantly higher intensities of both OC and ALP (Figure 6C) compared to the US or control cells and BMP2, which is indicative of increased osteoblast activity and differentiation compared to BMP2 stimulation.

## 3. Discussion

Determining the molecular mechanisms underlying osteoporosis is important. The current therapeutic products on the market mainly focus on decreasing osteoclast activity, and very few focus on increasing osteoblast activity, also called anabolic treatments. Teriparatide, a parathyroid hormone (1–34), is the only FDA-approved anabolic treatment. During its clinical trials, it increased total BMD compared to the placebo by 2–4% and was shown to have minor side effects [26]. However, this study was only done for 24 months, and long-term use has been linked to an increased risk of osteosarcoma in rats [27]. Two other potential anabolic therapeutics are abaloparatide and humanin. Abaloparatide is currently in phase 3 clinical trials, and humanin has been successful in increasing bone growth in varying models of in vivo and in vitro mouse and rat models [28,29]. While both are promising potential therapeutic products, there is still only one anabolic treatment that is approved by FDA. Osteoblasts build and create new bone; therefore, more research should be focused on increasing osteoblast activity versus decreasing osteoclast activity. Here, we showed that, as age increased, bone density remained unchanged in POA and decreased significantly in POP. As patients’ age increases, their BMD decreases, although POP still have lower BMD compared with patients not diagnosed with osteoporosis [7]. Ten years after menopause, women’s BMD is three times less than what it was prior to menopause. The average age for a woman to undergo menopause is 51, meaning that the average age of lower BMD would be around 61 [2]. Determining the BMD helped to confirm the extracted femoral heads were osteoporotic. Then we selected femoral heads closest to the trendline from the osteoporotic and osteoarthritic patient pool, where mature osteoblasts would be extracted. In order to determine and confirm that the cells extracted from the femoral heads were indeed osteoblasts, the cells were immunofluorescently stained for two osteoblast markers: OC and ALP. The positive staining for both unstimulated cells confirmed that we had successfully isolated a 99% pure and mature osteoblast culture from femoral heads. Cells extracted from POA had a significantly higher basal mineralization level compared to cells extracted from POP. CK2.3 has already been shown to increase mineralization in C2C12 cells and primary murine cells. Next, we wanted to determine the effect of CK2.3 on cells isolated from POP using the cells isolated from POA as a positive control. CK2.3 was observed to significantly increase mineralization to both unstimulated and BMP2-stimulated cells in POA, while only CK2.3 significantly increased mineralization compared to the control in the cells isolated from POP. CK2.3 also significantly increased expression of the osteoblast markers ALP and OC compared to the unstimulated and BMP2-stimulated cells. CK2.3 is hypothesized to bind to the interacting protein CK2 and inhibit its binding to a specific phosphorylation site on BMPRIa. CK2.3 induced a mineralization response in both sets of extracted cells, while BMP2 only induced a mineralization response in the osteoarthritic population, i.e., the population whose BMD did not decrease with increasing age. CK2.3 is hypothesized to act through the BMP pathway, and it has been shown to induce mineralization where BMP2 has not.

BMP2 is approved by the FDA for fracture healing in osteoporotic patients. However, it has recently been shown that prolonged treatment with BMP2 decreases the BMD in osteoporotic patients. Here, we showed that the BMD of POP decreased significantly in correlation with their increasing age [30]. In addition, the cells isolated from POP did not respond to BMP2 stimulation when assessed for mineralization potential. There is a variety of reasons as to why this could be occurring. For BMP2 to elicit its mineralization response, it must bind to its two dimerized receptors (a type Ia and a type II receptor). BMP2 will either bind preferentially to the type 1 receptor and recruit the constituently active type II receptor or it will bind to an already dimerized complex of receptors [31]. Previously, Turgeman et. al. had shown that human BMP2 (hBMP2) restored osteogenic potential of osteoporotic stem cells and that hBMP2 adenoviral vector increased osteogenic potential in a senile osteoporotic mouse model [32].

There are several limitations to this study. While a number of POP and POA were X-rayed and their BMD was quantified, only a select few had a cell population harvested from them. Further studies need to be done in order to verify the difference in BMP2 response between POP and POA. In addition, the means of BMD quantification through SPA is relatively novel, and while it is cost-effective and efficient, it does not provide the same level of detail as a density X-ray absorptiometry (DXA) scan. Only osteoblast-specific markers were investigated in this study because osteoblast activity and matrix deposition were of interest. In the future, it would be crucial to also investigate osteoclast activity and regulation as there is extensive crosstalk between osteoblasts and osteoclasts. This could be done through immunofluorescently staining for tartrate-resistant acid phosphatase (TRAP), osteoprotegerin (OPG), receptor activator of nuclear factor kappa-Β ligand (RANKL), and metalloproteinases (MMPs). Only femoral heads from female patients were utilized because both osteoporosis and osteoarthritis affect the female population more than the male population. In the future, we would like to investigate the effect of both BMP2 and CK2.3 in the male population to determine if this effect is mirrored.

In summary, we confirmed the diagnosis of extracted femoral heads through SPA. We also confirmed that we could successfully isolate mature osteoblasts from femoral heads through positive OC and ALP staining. Finally, we showed that BMD in POA remained unchanged as they age and that their extracted cells responded to both BMP2 and CK2.3. BMD significantly decreased in POP with increasing age, and while the corresponding extracted cells did not respond to BMP2, they did significantly respond to CK2.3. We also showed that there was a difference in BMP2 response between cell populations between the two skeletal diseases. It is clear that the BMP pathway is an important pathway with regard to osteoblast activity and understanding the pathology of osteoporosis. We observed a decrease in mineralization after BMP2 stimulation in cells extracted from POP, which would indicate a decrease in activation of the BMP pathway in POP. However, our novel peptide utilizes the BMP pathway in order to induce its osteogenic response; therefore, the signaling disparity of the BMP pathway in POP could be due to a decrease in BMP2 or a decrease in BMP2 stimulation. This discovery could lead to an explanation of the pathology of osteoporosis and why BMD is significantly lower in those diagnosed with osteoporosis. Currently, treating patients with BMP2 in order to promote fracture healing is pointless if those patients are diagnosed with osteoporosis and already have a decreased response to BMP2. Our peptide utilizes the BMP pathway in both skeletal diseases and induces an osteogenic response; therefore, it has greater therapeutic potential for osteoporosis, especially over BMP2. Further investigation could lead to a better understanding of what occurs on the molecular level in both skeletal diseases as well as the development of much-needed novel therapeutic products.

## 4. Materials and Methods

### 4.1. Subjects

Following institutional review board (IRB) exemption from Christiana Care Hospital, Newark, DE, (10 April 2013) human femoral heads were obtained after being extracted from patients undergoing hip arthroplasty surgery (DDD# 602228). The patients were diagnosed with osteoporosis or osteoarthritis (control). A total of 81 femoral heads were collected, of which 63 (aged 37–92) were from POP and 18 (aged 56–86) were from POA; all femoral heads were isolated from female patients.

### 4.2. X-Raying Femoral Heads

The femoral heads were X-rayed posterior to anterior with a Nomad Pro Veterinary Handheld X-ray System. A penny was positioned in the X-ray to verify that the distance between the handheld X-ray and the femoral head remained constant. After the radiographs were obtained, the pixel intensity (PI) of each femoral head was calculated.

### 4.3. Calculation of BMD for the Femoral Head

The radiographs were analyzed and measured in ImageJ. The PIs of two background regions of interest (ROI) were measured using the “Measure” function of ImageJ. They were then subtracted from the PI of the bone intensity ROI in order to obtain the BMD. PI is a measurement of a gray-level value on a scale of 0 (black) to 255 (white) and has been shown to correspond with bone density (mineralized) or BMD in several other studies [33,34,35]. This type of quantification of bone mineral density is called SPA as the X-ray penetrates through the sample in a single-photon ray and is reflected onto a detector [36].

### 4.4. Calculation of TMD for the Femoral Neck

Microcomputed tomography scans were taken and analyzed through a SCANCO MicroCT 35 device. A total of 1000 X-ray images were obtained at a range of 180° at different angles, with a filtered back-projection algorithm used to determine the “brightness value” of each voxel. The voxels’ “brightness value” was converted to density measurements through a conversion scale determined by several “brightness values” of metal rods of a known density. The trabecular volumes were manually defined, and the TMD reported is the averaged density of voxels within that particular ROI. A trabecular ROI from the image stack was defined by manually contouring the trabecular bone roughly for an irregular anatomic region a few pixels from the cortical bone for 16 slides and interpolating that to 231 slides. The microCT scans were treated with a Gaussian filter to remove noise, and the ROIs were then subjected to auto thresholding, with the threshold for trabecular bone to be 35% maximal brightness. Several standard morphological measures of cortical and trabecular bone were reported for the contoured trabecular and cortical ROIs. TMD measured the averaged density of all voxels, including voids within the volume defined by the contours (or ROI).

### 4.5. Isolation of Primary Osteoblasts

In a sterile environment, femoral heads were selected from the trendline on the BMD versus age graph (Figure 1E) and then sliced down the midsagittal plane with an Arbor cut-off saw (Drill Master, 14 inch HP cut-off saw) or DREMEL 4000. Bone fragments of cancellous bone were extracted from the interior surface of the bone, washed once with 1X phosphate-buffered saline (PBS), and digested with a DMEM/collagenase (Dulbecco’s Modification of Eagles Medium, Corning; Collagenase Type II, Worthington) solution for two days. The cellular suspension was filtered using a 70 µm cell strainer (BD Falcon), centrifuged to pellet, resuspended in fresh DMEM with no collagenase solution, and plated in a T25 flask. Cells were grown for seven days without a media change to ensure cell adhesion to the bottom of the flask. After the seventh day, fresh DMEM media was supplemented to the cells every four days in order to promote cell growth.

### 4.6. Immunostaining

Cells were isolated from three female osteoporotic patients whose ages were 60, 73, and 76. The cells were seeded in a 24-well plate at a density of 1 × 10^5^ cells/cm^2^ per well. Once 90% confluent cells were serum-starved overnight and treated with 100 nm CK2.3 or 40 nm BMP2 or left unstimulated. After five days of treatment, cells were washed with 1X PBS and then fixed with acetone and methanol. The samples were fluorescently labeled for one hour at room temperature for rabbit polyclonal IgG osteocalcin as a 1:200 dilution (200 μg/mL, Santa Cruz Biotechnology, Dallas, TX, USA), which was followed by Alexafluor chicken antirabbit as a 1:500 dilution (200 μg/mL, Life Technologies, Carlsbad, Ca, USA) and goat polyclonal IgG alkaline phosphatase as a 1:200 dilution (200 μg/mL, Santa Cruz Biotechnology), followed by Alexafluor 568 donkey antigoat IgG as a 1:500 dilution (200μg/mL, Life Technologies, Carlsbad, CA, USA). All antibodies were diluted in a 3% bovine serum albumin (BSA) solution. Bisbenzimide (Sigma-Aldrich, St. Louis, MO, USA Hoechst dye No. 33258, dissolved in H_2_O) was used as a nuclear stain for two and a half minute incubation. The coverslips were mounted using Airvol, as previously described [37,38]. Images were taken on Zeiss Axiophot with a 20 × objective (Fluor, Zeiss, Germany) and analyzed in ImageJ (NIH, Bethesda, MD, USA).

### 4.7. von Kossa Assay

Once cells were grown to confluency in a T25 flask, they were split onto a 24-well plate at a density of 1 × 10^5^ cells/cm^2^. Once 90% confluent, cells were serum-starved overnight and treated with either 100 nM CK2.3 or 40 nM BMP2 or left unstimulated (control). These concentrations were determined to be optimal for promoting osteogenesis [22]. After five days, the assay was conducted as previously described [22]. Briefly, cells were washed with 1X PBS, fixed with 4.4% (w/v) paraformaldehyde (PFA) for 15 minutes, and assayed using 5% (w/v) silver nitrate (Sigma Aldrich, St. Louis, MO, USA) solution in order to determine calcium deposits or mineralization. Ten images were taken of each well and quantified using ImageJ (NIH, Bethesda, MD, USA). Images were converted to 8 bits, and a threshold was set to the control and subsequently used for all treatments within an individual experiment. The surface area stained with silver (and represented mineralization) was quantified using the “Analyzing Particles” function of ImageJ.

### 4.8. Immunostaining Quantification

Immunofluorescent images were quantified using ImageJ. Briefly, images were converted to 8 bits, and the threshold was then adjusted to the 2nd or negative control to eliminate nonspecific staining. Once converted, the images were black and white, which made it easier to calculate pixel intensity. Pixel intensity was calculated through the measure function of ImageJ and was averaged for BMP2-stimulated, CK2.3-stimulated, and unstimulated cells. Fluorescent staining intensity has been shown to be equivalent to the pixel intensity measured in ImageJ [39].

### 4.9. Statistical Analysis

BMD data were analyzed through linear regression analysis, and outliers were removed through Chauvenet’s criterion. von Kossa data were analyzed through an ANOVA with a Tukey–Kramer post hoc test. Outliers were removed through Chauvenet’s criterion, and error bars depict standard error of the mean.

## Abbreviations

POA	patients diagnosed with osteoarthritis
POP	patients diagnosed with osteoporosis
OA	osteoarthritis
OP	osteoporosis
BMD	bone mineral density
DXA	density X-ray absorptiometry
SPA	single-photon absorptiometry
